# Knowledge of health care workers and ability of healthcare facilities in preventing of Ebola virus diseases/lassa fever in Benin

**DOI:** 10.1186/2047-2994-4-S1-P4

**Published:** 2015-06-16

**Authors:** AK Aissi, AT Ahoyo, CB Orou Yorou, L Toko, L Affovehounde, MT Gounoungbe, C Glele Kakaï, DA Kinde Gazard

**Affiliations:** 1 Direction Nationale la Santé Publique, Benin; 2 ministère de la Santé, Cotonou, Benin

## Introduction

Benin republic, because of its geographical location, is threatened by the epidemic of the Ebola virus disease (EVD) that continues to plague in West Africa.

## Objectives

Analyze the capacity of the health system in Benin relative to the axis of prevention of Viral Hemorrhagic Fevers.

## Methods

Cross-sectional evaluative study from january to february 2015. Self-administered questionnaire to 418 health workers in all categories. They were from 52 health facilities identified in 08 of the 12 departments of Benin. Semi-structured interviews with health authorities responsibles of epidemiological and hospital hygiene matters. Direct observation of systems and existing procedures was made.

## Results

91% of workers know the early signs of EVD. 60% follow the news concerning Ebola in Africa. 28% don't know the distinction between the EVD and the Lassa fever which an epidemic has been recently controlled successfully in northern Benin. 67% know the EVD transmission modes. 42% didn't know the incubation period for EVD, and 81% know how to identify a suspected case. 87% are convinced that there is no cure treatment even in african medicine. 94% admit that awareness raising currently underway on Ebola improve the awareness in applying hospital hygiene guidelines. 91%, 43%, 42% and 39% respectively know that the Ebola virus may be neutralized by bleach, alcohol at 70°, ultraviolet light and formol. 81% assume that hand hygiene solutions are an effective way to prevent and control infections, but its supply in care services is uncommon or irregular. 51% of responsibles think that the alcohol-based handrub dispensers are expensive and in absence of subsidies or grants, their purchase could lead to significant extra costs for patients. The Methods of treatment of biomedical waste are not performing with a disparity depending on the size of care facilities.

## Conclusion

The capacities of the hospital system in Benin are underperforming for the prevention of EVD and Lassa fever. Infection prevention and control strategies must be implemented in the short term for the safety of patients and staff.

## Disclosure of interest

None declared.

